# Nonreciprocal vortex isolator via topology-selective stimulated Brillouin scattering

**DOI:** 10.1126/sciadv.abq6064

**Published:** 2022-10-19

**Authors:** Xinglin Zeng, Philip St.J. Russell, Christian Wolff, Michael H. Frosz, Gordon K. L. Wong, Birgit Stiller

**Affiliations:** ^1^ Max Planck Institute for the Science of Light, Staudtstr. 2, 91058 Erlangen, Germany.; ^2^ Center for Nano Optics, University of Southern Denmark, Campusvej 55, DK-5230 Odense M, Denmark.; ^3^ Department of Physics, Friedrich-Alexander University, Staudtstr. 2, 91058 Erlangen, Germany.

## Abstract

Optical nonreciprocity, which breaks the symmetry between forward and backward propagating optical waves, has become vital in photonic systems and enables many key applications. So far, all the existing nonreciprocal systems are implemented for linearly or randomly polarized fundamental modes. Optical vortex modes, with wavefronts that spiral around the central axis of propagation, have been extensively studied over the past decades and offer an additional degree of freedom useful in many applications. Here, we report a light-driven nonreciprocal isolation system for optical vortex modes based on topology-selective stimulated Brillouin scattering (SBS) in chiral photonic crystal fiber. The device can be reconfigured as an amplifier or an isolator by adjusting the frequency of the control signal. The experimental results show vortex isolation of 22 decibels (dB), which is at the state of the art in fundamental mode isolators using SBS. This device may find applications in optical communications, fiber lasers, quantum information processing, and optical tweezers.

## Teaser

Optical vortex isolation is enabled by topology-selective stimulated Brillouin scattering in chiral photonic crystal fiber.

## INTRODUCTION

Breaking two-way optical symmetry so as to achieve nonreciprocal isolation is crucial in many laser, amplifier, optical communications, and sensing systems (*1*). In particular, it is of great importance in all-optical signal routing, protecting lasers from disruptive back-reflections, and reducing multipath interference in optical communications. Moreover, isolators generally improve the performance of optical systems through suppression of unwanted interference, interdevice interactions, and channel cross-talk. Most conventional optical isolators are based on the Faraday effect in magneto-optical materials (*2*, *3*). Alternative approaches—such as optical isolation through the Kerr effect (*4*, *5*), optomechanically induced transparency (*6*, *7*), spatial-temporal modulation (*8*, *9*), and stimulated Brillouin scattering (SBS) (*10*–*12*)—have also been proposed and demonstrated, although only for linearly or randomly polarized LP_01_-like fundamental modes.

Circularly polarized vortex beams or modes have been extensively studied in recent years, in connection with applications in quantum and classical communications (*13*, *14*), optical nanomanipulation (*15*, *16*), and quantum information processing (*17*). As a result, devices such as vortex generators, lasers, and signal amplifiers have been demonstrated and are in great demand (*18*). A device that is, so far, missing is a vortex isolator, which is essential for high-power vortex laser systems and quantum manipulations.

Here, we report the first example of a reconfigurable light-driven nonreciprocal isolator for circularly polarized vortex modes based on topology-selective SBS in chiral photonic crystal fiber (PCF). In recent years, chiral PCF (*19*), which offers a unique platform for studying the behavior of light in chiral structures that are infinitely extended in the direction of the twist, has been shown to robustly preserve optical modes carrying circular polarization states and optical vortices over long distances, allowing investigation of nonlinear processes in the presence of chirality (*20*, *21*). We report isolation of vortex modes through topology-selective SBS in chiral PCFs with threefold rotational symmetry (C_3_ PCF) and sixfold rotational symmetry (C_6_ PCF). In particular, angular momentum conservation dictates that the topological charge and spin of the backward Brillouin signal are opposite to those of the pump. The experiments show isolation factors higher than 22 dB for two different vortex orders, which are comparable with the best SBS-related optical isolators for fundamental modes (*22*). The isolation remains nearly constant within 2 dB over a 35-dB dynamic range of signal power, indicating excellent optical linearity. Switching between nonreciprocal isolation and amplification can be simply achieved by down- or up-shifting the control signal frequency by the Brillouin frequency. We also develop an analytical theory for the dynamics of nonreciprocal SBS in chiral PCF and achieve good agreement with experimental measurements.

## RESULTS

### Topology-selective SBS in chiral PCF

*N*-fold rotationally symmetrical (symmetry class C*_N_*) chiral PCFs support helical Bloch modes (HBMs) (*23*), whose *m-*th order azimuthal harmonics carry optical vortices with azimuthal order 𝓁_A_^(*m*)^ = 𝓁_A_^(0)^ + *Nm*, where 𝓁_A_^(*m*)^ is the number of complete periods of phase progression around the azimuth for fields expressed in cylindrical components and 𝓁_A_^(0)^ is the principal azimuthal order. Note that 𝓁_A_ is always an integer and is robustly conserved. In chiral PCF, it is found, both experimentally and by numerical modeling, that the fields are almost perfectly circularly polarized. In this case, the cylindrical transverse electric fields can be expressed in Cartesian components simply by multiplying them with a rotation matrix, leading to an expression that links the azimuthal order 𝓁_A_^(*m*)^ to the topological charge 𝓁_T_^(*m*)^: 𝓁_A_^(*m*)^ = 𝓁_T_^(*m*)^ *+ s*, where spin *s* = +1 denotes a left circular polarization state. Here, we use the shorthand [𝓁_T_, *s*] to denote the parameters of the circularly polarized vortex–carrying HBMs, where, for ease of notation, 𝓁_T_ = 𝓁_T_^(0)^ is defined as the principal topological order. In chiral PCF, HBMs with equal and opposite values of 𝓁_T_ are generally nondegenerate in index, i.e., topologically birefringent, while modes with opposite spin but the same value of 𝓁_T_ are weakly birefringent (*23*).

**Fig. 1.**
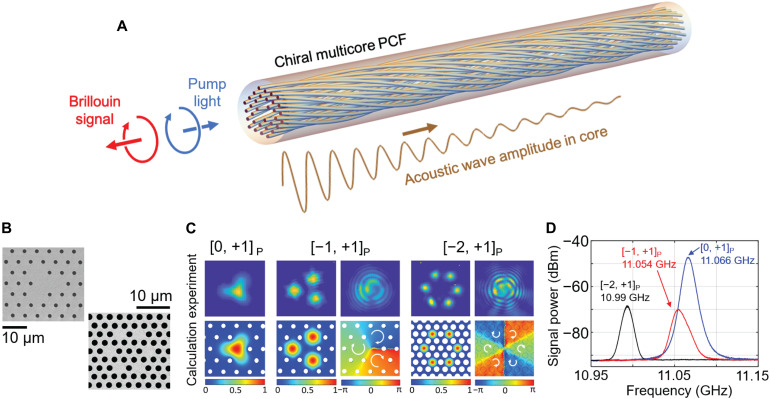
Topology-selective Brillouin scattering in chiral PCF. (**A**) Conceptual view of SBS in chiral threefold rotationally symmetric PCF. The circular arrows indicate both the spin and the direction of azimuthal phase progression, which are preserved during SBS. As a result, pump and Stokes signals interact only when the signs of 𝓁_T_ and *s* are reversed relative to the beam directions. (**B**) SEMs of the C_3_ PCF and C_6_ PCF used in the experiments. (**C**) Experimentally measured and numerically calculated near-field distributions for [𝓁_T_, *s*]_P_ = [0, +1] and [−1, +1] in C_3_ PCF and [𝓁;_T_, *s*]_P_ = [−2, +1] in C_6_ PCF. The subscript P denotes pump wave. Interference patterns between the vortex-carrying HBMs and a divergent Gaussian beam are shown in the top right of each higher-order HBM. The calculated polarization and phase distributions are shown in the bottom right of each higher-order HBM. The measured losses of [0, ±1] and [±1, ±1] modes in C_3_ PCF are 0.013 and 0.017 dB/m, and the loss of the [±2, ±1] modes in C_6_ PCF is 0.04 dB/m. The index difference is ~8 × 10^−4^ between [+1, ±1] and [−1, ±1] modes and ~7 × 10^−4^ between [+2, ±1] and [−2, ±1] modes, and the circular birefringence at fixed 𝓁_T_ is ~6 × 10^−6^ in C_3_ PCF and ~2 × 10^−5^ in C_6_ PCF. (**D**) Spontaneous Brillouin spectra generated by pumping C_3_ PCF (at 1550 nm) with [0, +1]_P_ (blue) and [−1, +1]_P_ (red) modes and C_6_ PCF with the [−2, +1]_P_ (black) mode.

Topology-selective SBS in chiral PCF is a process in which a forward pump (P) mode scatters strongly into a backward Stokes (S) mode when [𝓁_T_, *s*]_P_ = −[𝓁_T_, *s*]_S_ but shows no interaction when [𝓁_T_, *s*]_P_ = [𝓁_T_, *s*]_S_, as illustrated in Fig. 1A. The fields of the forward (pump) and backward (Stokes) HBMs can be written as

$$\begin{array}{c} E_{P}(r,t)=A_{P}(z)a(x,y)u_{P}\;e^{i(\beta_{P}z+\ell_{\text{TP}}\phi )}e^{-i\omega_{P}t}\\ E_{S}(r,t)=A_{S}(z)a(x,y)u_{S}^{*}\;e^{-i(\beta_{S}z+\ell_{\text{TS}}\phi )}e^{-i\omega_{S}t} \end{array}$$ (1)

where $u_{k}=(u_{kx},u_{ky})=(1,{\text{is}}_{k})/\sqrt{2}$ is a complex unit vector ($u_{k}\cdot u_{k}^{*}=1$) representing the polarization state, ℓ_T*k*_ is the topological charge, subscript *k* = P or S denotes pump or Stokes, *A_k_*(*z*) is a slowly varying amplitude, *a*(*x*,*y*) is the scalar modal field distribution (assumed identical for both modes), β*_k_* is the propagation constant, and ω*_k_*/2π is the optical frequency. The vortex-free acoustic density wave excited by Brillouin scattering may similarly be written as

$$\rho (r,t)=\rho_{m}a_{q}(x,y)e^{i(qz-\Omega t)}$$ (2)

where *q* and Ω/2π are the acoustic wave vector and frequency. The optoacoustic overlap is then proportional to the integral over the transverse cross section of

$$(E_{P}\cdot E_{S}^{*})\rho *=A_{P}A_{S}^{*}\rho *a^{2}a_{q}e^{i(\ell_{\text{TP}}+\ell_{\text{TS}})\phi }(u_{P}\cdot u_{S})e^{i(\beta_{P}+\beta_{S}-q)z-i(\omega_{P}-\omega_{S}-\Omega )t}$$ (3)

which is nonzero only if ℓ_TP_ = − ℓ_TS_ and **u**_P_ · **u**_S_ = 1, i.e., *s*_P_ = –*s*_S_. The Brillouin gain is maximized when both energy and momentum are conserved, i.e., Ω = ω_P_ − ω_S_ and *q* = β_P_ − β_S_.

### Noise-initiated SBS measurement

Two different chiral PCFs were used in the experiments. The first had a threefold rotational symmetry (C_3_ PCF) and a twist period of 5 mm (*24*), and the second had a sixfold rotational symmetry (C_6_ PCF) and a twist period of 7.2 mm. The preforms were constructed by the standard stack-and-draw process, and the fibers were drawn from a spinning preform. Scanning electron micrographs (SEMs) of the two PCFs are shown in Fig. 1B. The hollow channel diameter *d* and interchannel spacing Λ were 1.6 and 5.16 μm for the C_3_ PCF, respectively, and 2 and 2.98 μm for the C_6_ PCF. Figure 1C shows measured near-field intensity profiles, alongside numerical simulations, for [0, +1]_P_ and [−1, +1]_P_ HBMs after propagation along 200 m of C_3_ PCF and the [−2, +1]_P_ HBM after propagation along 200 m of C_6_ PCF. For each vortex mode, the top right shows the spiral fringe pattern formed by interference with a divergent Gaussian beam; the bottom shows the corresponding calculated phase distributions. According to the helical Bloch theory in the previous section, any deviations from integer values of topological charge are caused by the polarization state not being exactly circular. In the measurement, the modulus of the Stokes parameter, |S_3_|, is higher than 0.98 at the output of both PCFs, showing very good preservation of circular polarization state, and yielding an integer-valued topological charge, even after 200 m of propagation.

Figure 1D shows a heterodyne measurement of the backscattered Stokes signal, formed by mixing with a local oscillator to produce an electrical beat note in the gigahertz domain (for details, see section S1). The Brillouin frequency shifts were 11.066 GHz for [0, ±1]_P_ and 11.054 GHz for [±1, ±1]_P_ in C_3_ PCF and 10.99 GHz for [±2, ±1]_P_ in C_6_ PCF. The peaks in the spectrum correspond to acoustic modes that overlap strongly with, and phase match to, the optical modes. Numerical calculations using finite element modeling show good agreement with the experimental results, predicting peaks at 11.085, 11.063, and 11.027 GHz (the associated acoustic modes are shown in section S2). Compared to PCFs with high air-filling fractions and micrometer-scale cores, which guide several different types of acoustic core modes and produce a multipeaked Brillouin spectrum (*25*), the relatively low air-filling fraction and large core diameter of the two chiral PCFs produce a Brillouin frequency shift close to that of bulk glass: 2*n*_s_*v*_L_/λ_p_= 11.12 GHz, where *n*_s_ is the refractive index of silica at the pump laser wavelength λ_p_ and *v*_L_= 5971 m/s is the longitudinal acoustic phase velocity.

**Fig. 2.**
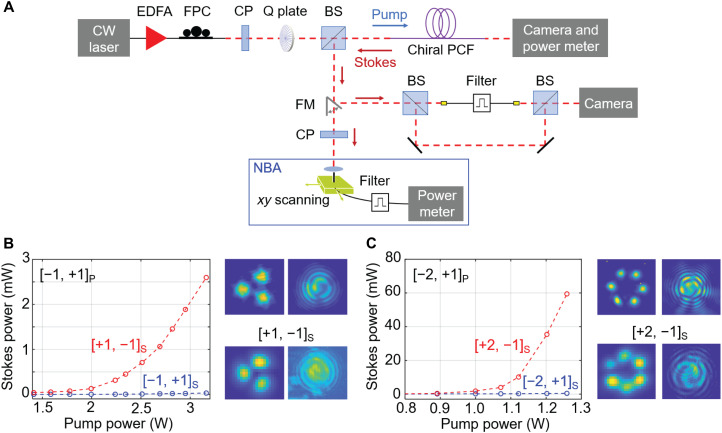
Noise-initiated topology-selective SBS in chiral PCF. (**A**) Experimental setup for measuring the Stokes signal with vortex pumping. CW, continuous wave; FPC, fiber polarization controller; CP, circular polarizer; FM, flip mirror; Q plate, an optical device that can generate vortex-carrying light beams from a circularly polarized Gaussian beam. (**B**) Stokes signal power, polarization state, mode profile, and topological charge for pumping C_3_ PCF with a [−1, +1]_P_ mode. (**C**) Same as (B) for pumping C_6_ PCF with a [−2, +1]_P_ mode. The broad bandwidth (~40 MHz) of the spontaneous Stokes signals somewhat obscures the Stokes interference patterns, while the less noisy pump patterns are much cleaner.

We next increased the pump power above threshold so as to reach the SBS regime, when the polarization states, mode profiles, and topological charges of the much stronger Stokes signals could be more easily and precisely measured. Only the measurements for vortex-carrying ([−1, +1]_P_ and [−2, +1]_P_) HBM pumps are presented here (for completeness, measurements for [0, ±1]_P_ are available in section S3). Figure 2A shows the experimental setup (for more details, see Materials and Methods). Figure 2 (B and C) shows the power dependence of the Stokes signal for [−1, +1]_P_ in C_3_ PCF and [−2, +1]_P_ in C_6_ PCF. As already mentioned, the spin and topological charge of the Stokes signal are opposite in sign to that of the pump (see interferometric patterns in Fig. 2, B and C). The robustness of spin and topological charge preservation in chiral PCF was further confirmed by measurement of the Stokes parameter S_3_ of the Stokes signal for [−1, +1]_P_ and [−2, +1]_P_. The measured value of |S_3_| was greater than 0.96 in all cases (for details, see section S4).

### SBS gain coefficient measurement

**Fig. 3.**
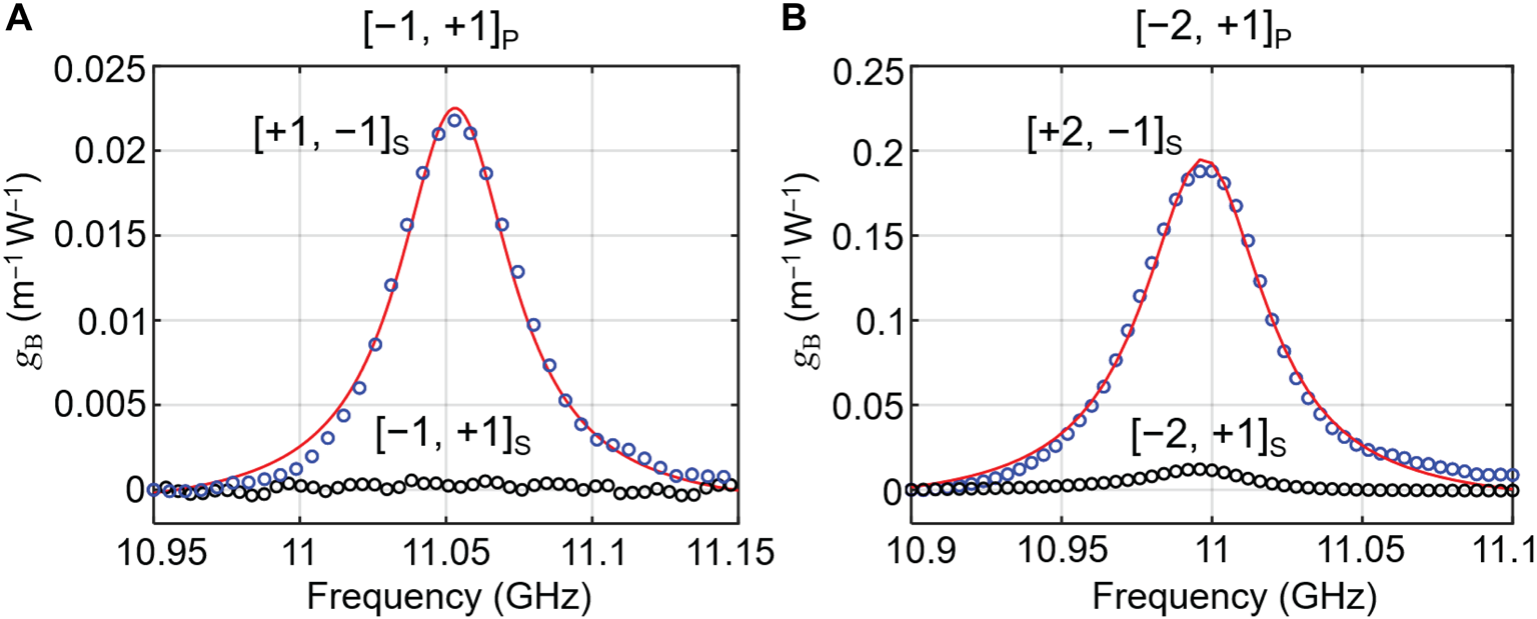
Brillouin gain measurement in chiral PCF. (**A**) Frequency dependence of the Brillouin gain *g*_B_ in C_3_ PCF pumped by a [−1, +1]_P_ mode. The circles are measured data, and the red lines are Lorentzian fits. (**B**) Same as (A) but for C_6_ PCF pumped by the [−2, 1]_P_ mode.

We next measured the Brillouin gain coefficients *g*_B_ for vortex modes in C_3_ and C_6_ PCF, both 200 m long. The amplified Stokes signal takes the well-known form (*26*)

$$P_{S}(0)=P_{S}(L)\text{exp}(g_{B}P_{P}(1-e^{-\alpha L})/\alpha -\alpha L)$$ (4)

where α is the fiber loss (0.017 dB/m for [±1, ±1] modes in C_3_ PCF and 0.04 dB/m for [±2, ±1] modes in C_6_ PCF), *L* is the fiber length, and *P*_P_ is the pump power. Details of the experimental setup are available in section S5. Figure 3 plots *g*_B_, estimated from the experimental measurements using Eq. 4, as a function of pump-seed frequency difference at a pump power of 0.8 W and a Stokes seed power of 10 mW. As expected, the gain is substantial only when the pump and seed have opposite topological charge and spin, reaching peak values of 0.022 m^−1^ W^−1^ for [−1, +1]_P_ and [+1, −1]_S_ in C_3_ PCF and 0.185 m^−1^ W^−1^ for [−2, +1]_P_ and [+2, −1]_S_ in C_6_ PCF. These two measured peak gain coefficients are close to the theoretical values of 0.032 and 0.162 m^−1^ W^−1^, calculated using the theoretical expression for the line-center gain coefficient, taking account of the optoacoustic overlap (*27*). Note that since the topological charge of the Stokes signal is opposite in sign to that of the pump, the identical acoustic mode is excited for both signs of 𝓁_*T*P_. For completeness, the measured gain spectra for [0, +1]_P_ and [0, −1]_S_ modes in C_3_ PCF are available in section S5, showing peak gain coefficients of 0.169 m^−1^ W^−1^.

### Reconfigurable light-driven vortex isolator

**Fig. 4.**
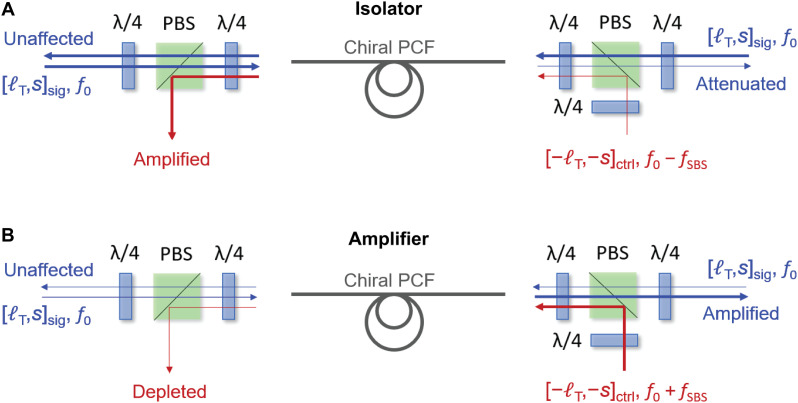
Schematic diagrams of isolator and amplifier. (**A**) Nonreciprocal optical isolator. The control signal is combined with, or separated from, the signal using PBSs. When a backward control wave [−𝓁_T_, −*s*]_ctrl_ with frequency *f*_0_ − *f*_SBS_ is launched, the forward signal [𝓁_T_, *s*]_sig_ is highly attenuated, while the backward-propagating signal [𝓁_T_, *s*]_sig_ is unaffected. (**B**) When the backward control wave frequency is changed to *f*_0_ + *f*_SBS_, the forward signal [𝓁_T_, *s*]_sig_ is amplified, while the backward-propagating signal [𝓁_T_, *s*]_sig_ is unaffected.

Next, we used the setup to demonstrate a nonreciprocal vortex isolation system (Fig. 4). Each circularly polarized vortex–carrying beam passes through two λ/4 plates placed on opposite sides of a polarizing beam splitter (PBS), permitting the polarization state in each path to be sequentially converted from circular to linear and from linear to circular. Where necessary, we denote control parameters with the subscript “ctrl” and signal parameters by subscript “sig.” When a signal (parameters [𝓁_T_, *s*]_sig_) at frequency *f*_0_ is launched into the fiber, along with a counterpropagating control wave (parameters [−𝓁_T_, −*s*]_ctrl_) at frequency *f*_0_ − *f*_SBS_, power is transferred from the signal to the control wave, and the signal is attenuated. In contrast, a counterpropagating signal with parameters [𝓁_T_, *s*]_sig_ at frequency *f*_0_ is unaffected. If the control wave frequency is changed to *f*_0_ + *f*_SBS_, keeping all other parameters constant, then the power is transferred from the control to the signal, which is amplified. Once again, a counterpropagating signal (parameters [𝓁_T_, *s*]_sig_) at frequency *f*_0_ is unaffected. The system can thus be conveniently configured as a nonreciprocal amplifier or attenuator by adjusting the control signal frequency.

**Fig. 5.**
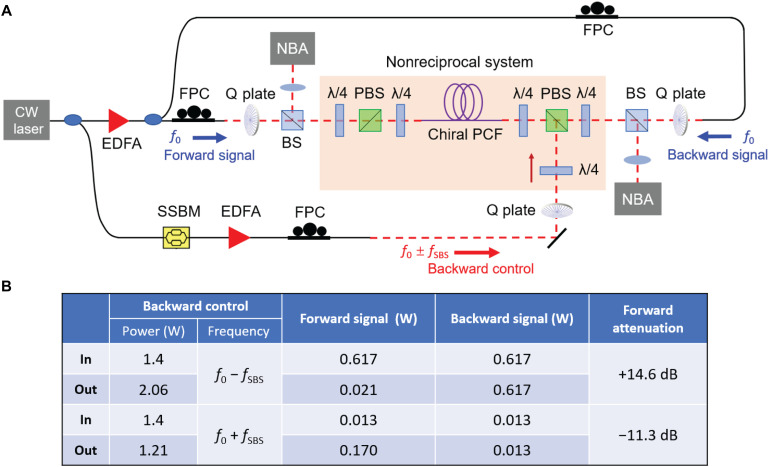
Experimental setup and measurements. (**A**) Schematic diagram of the reconfigurable light-driven vortex isolator. The chiral fiber was 200 m long in all of the experiments. (**B**) Nonreciprocal isolation and amplification measurements for [−1, +1] modes. The forward signal waves are strongly depleted (top two rows) or amplified (bottom two rows) by backward control waves, while the backward signal wave is unaffected.

**Fig. 6.**
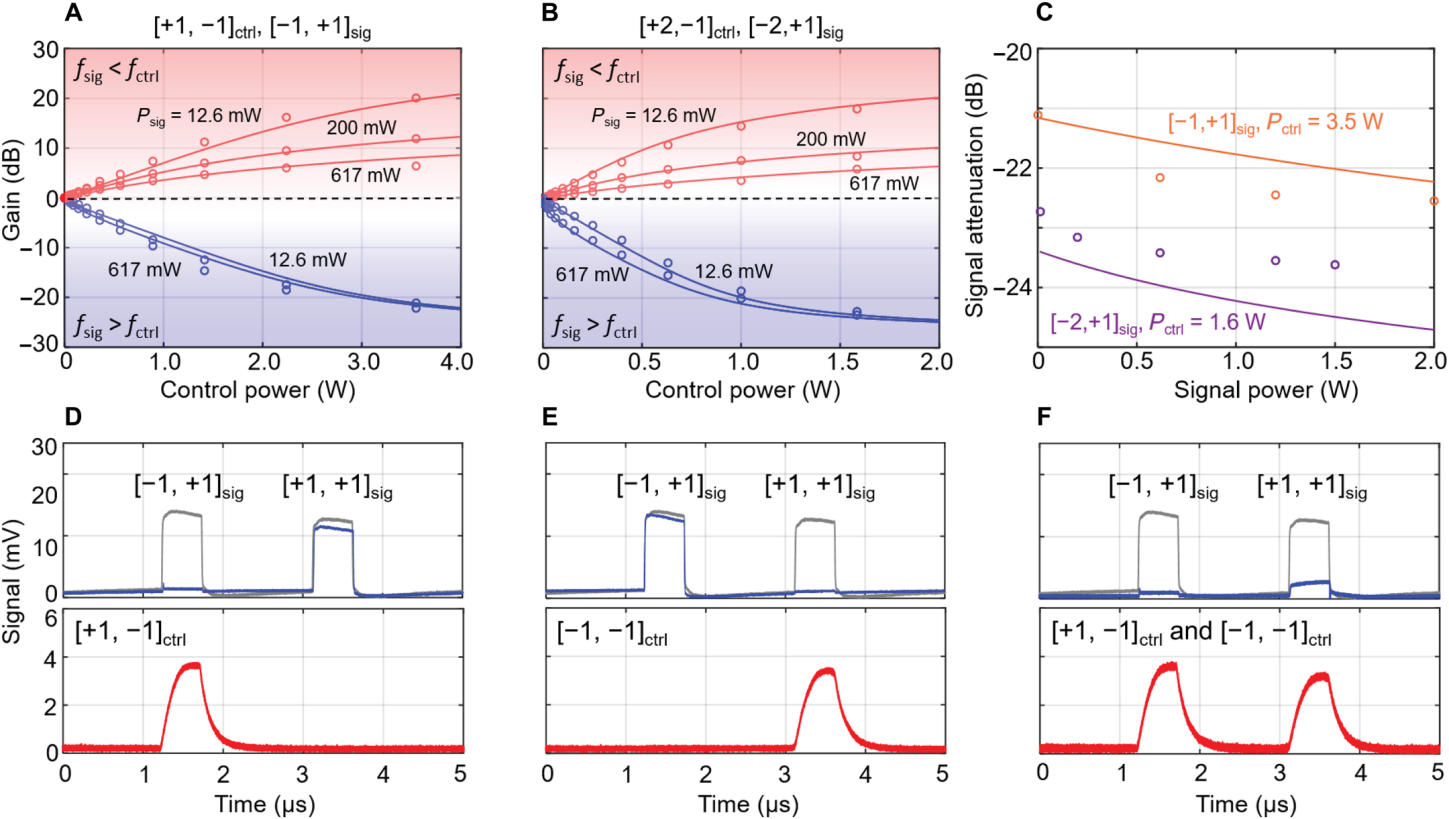
Nonreciprocal attenuation and amplification. (**A**) Dependence of [−1, +1]_sig_ gain on [+1, −1]_ctrl_ power for *f*_sig_ < *f*_ctrl_ (top red-shaded region) and *f*_sig_ > *f*_ctrl_ (bottom blue-shaded region). *P*_sig_, signal power; *P*_ctrl_, control power. (**B**) Same as (A) but for [−2, +1]_sig_ and [+2, −1]_ctrl_ modes. (**C**) Dependence of signal attenuation on signal power. The circles are experimental data points, and the curves are theoretical predictions based on Eq. 5. (**D** to **F**) The temporal-multiplexed pulses at the input (gray) and output (blue) of isolator and the control wave at the output of isolator (bottom), with the control wave assigned to be (D) [+1, −1]_ctrl_, (E) [−1, −1]_ctrl_, and (F) both. Two multiplexed pulses are separately encoded with [−1, +1]_sig_ and [+1, +1]_sig_ modes.

Figure 5A shows the experimental setup for observing nonreciprocity (see Materials and Methods for more details). Figure 5B tabulates the measured power in the transmitted forward and backward signals in each case, for example, a forward [−1, +1]_sig_ mode is strongly attenuated by a backward [+1, −1]_ctrl_ mode in C_3_ PCF, while the backward [−1, +1]_sig_ mode is unaffected. The dependence on control power of the attenuation and amplification of [−1, +1]_sig_ and [−2, +1]_sig_ modes is explored in Fig. 6 (A and B). For a signal power of 617 mW, the isolation is 22.2 dB for the [−1, +1]_sig_ mode (left figure, blue) and 23.4 dB for the [−2, +1]_sig_ mode (right figure, blue). By switching the frequency of the control signal from *f*_0_ − *f*_SBS_ to *f*_0_ + *f*_SBS_, nonreciprocal amplification factors of 21 dB for the [−1, +1]_sig_ mode (left-hand figure, red) and 18 dB for the [−2, +1]_sig_ mode (right-hand figure, red) were obtained, keeping the signal power at 12.6 mW.

It is notable that the isolation saturates with increasing control light power (Fig. 6, A and B). We attribute this to the onset of second-order SBS when the first Stokes signal becomes strong. This is aided by second harmonic distortion in the single-sideband modulator (SSBM), which generates a signal at (*f*_0_ − 2*f*_SBS_) that is ~27 dB weaker than the first harmonic. This signal is then Fresnel-reflected at the input face of the fiber, thereby seeding the second Stokes signal. The well-known coupled wave theory of SBS, based on slowly varying amplitudes, can be used to treat this case (*28*), leading to the coupled power equations

∂PP∂z=−(α+gBPS1)PP∂PS1∂z=(α+gB(PS2−PP))PS1∂PS2∂z=(−α+gBPS1)PS2 (5)

where *P*_P_ is the pump power, *P*_S1_ is the first (backward) Stokes power, and *P*_S2_ is the second (forward) Stokes power. The exponential power attenuation rate is α m^−1^, and *g*_B_ is the effective Brillouin gain in units of per meter per watt. Experimental estimates of *g*_B_ are 0.022 m^−1^ W^−1^ for the C_3_ PCF and 0.185 m^−1^ W^−1^ for the C_6_ PCF. Although the Brillouin frequency shifts for P-to-S1 and S1-to-S2 conversion are not strictly the same, the difference is only 1 part in 10^4^, so it can be neglected. As shown in Fig. 6 (A and B), numerical solutions of Eq. 5 are in good agreement with the measurements, confirming that the saturation is caused by cascaded SBS.

In applications such as optical communications and all-optical signal processing, it is vital that isolation is effective over a wide range of different signal powers. The chiral fiber performs very well, offering ~22 dB of isolation within ±1 dB for the [−1, +1]_sig_ mode (orange circles in Fig. 6C) over a 35-dB range of signal power at a control power of 3.55 W. The same is true for the [−2, +1]_sig_ mode (purple circles in Fig. 6C) at a control power of 1.59 W. The measurements are in reasonable agreement with theoretical calculations, as shown by solid curves in Fig. 6C.

Isolators based on chiral PCF have the advantage of scalability and can be used for nonreciprocal attenuation of multiple vortex beams, which could be useful in multiplexing systems. As a proof-of-concept experiment, we multiplexed two modes with [−1, +1]_sig_ and [+1, +1]_sig_ in the isolator. The experimental setup is shown in section S7, and the results are shown in Fig. 6D. The gray-colored pulses are multiplexed signals in the forward direction without backward control. The left pulse is encoded with [−1, +1]_sig_ and the right one with [+1, +1]_sig_. Both pulses have an average power of 617 mW and are synchronized and time division multiplexed. The pulse widths are 500 ns, and the smallest time gap between two multiplexed pulses was measured to be 1.89 μs at the output of isolator. By launching a 1.4-W continuous-wave (CW) control wave in the [+1, −1]_ctrl_ mode, the left [−1, +1]_sig_ pulse is depleted, but the right [+1, +1]_sig_ pulse is almost unaffected (we attribute the tiny depletion to slight mode cross-talk), as shown in Fig. 6D (blue trace). Meanwhile, the control wave is amplified within the time window corresponding to the [−1, +1]_sig_ pulse. Similarly, when the control wave carries only the [−1, −1]_ctrl_ mode, the right signal pulse is depleted, while the left pulse remains almost unchanged, and the control wave is amplified within the time window corresponding to the right signal pulse, as shown in Fig. 6E. Last, Fig. 6F shows that both signal pulses are depleted when a control wave with both modes is launched. In the measurement, the pulsed signals in the backward direction are unaffected. This demonstration of selective isolation of different vortex modes confirms again the robustness of topology-selective SBS.

## DISCUSSION

Efficient light-driven optical isolators for circularly polarized HBMs can be realized by topology-selective SBS in chiral *N*-fold rotationally symmetric multicore PCF. HBMs with a principal topological charge up to the nearest integer below |*N*/2| can be guided and isolated. Nonreciprocal behavior follows because Brillouin gain only exists when the Stokes and pump signals have equal and opposite topological charge and spin. Isolation factors greater than 22 dB are obtained over a 35-dB dynamic range of input signal. Nonreciprocal vortex amplification can be achieved by using a control signal with frequency *f*_SBS_ above the vortex signal. Although the working bandwidth is limited by the Brillouin gain linewidth, isolation over a much wider bandwidth is possible if the control wavelength is tuned in synchronism with the signal wavelength.

The chiral PCFs used in the experiments reported here (twist periods of 5 and 7.2 mm) are able to robustly preserve circular polarization state and vorticity over long distances. In general, the twist rate must be high enough to produce a circular birefringence that overcomes inevitable deviations from perfect *N*-fold rotational symmetry in the PCF structure. Although, in principle, a perfect untwisted C*_N_* PCF will support pure circularly polarized vortex modes, in practice, these modes are highly sensitive to inevitable small structural imperfections that vary along the fiber, causing interpolarization coupling (*20*, *29*).

Note that imperfect launch optics can result in weak excitation of unwanted vortex modes. The consequences of this can be clearly observed in Fig. 3B, where a tiny gain is still observed when the pump and seed have the same topological charge and spin (black circles). Although this will impair the effectiveness of vortex isolation, it can be suppressed by optimizing the launch optics.

We note that, in principle, the pump and Stokes waves can have different values of topological charge, but only if the acoustic mode provides the missing angular momentum, i.e., it is itself a vortex mode. Here, however, there is no angular momentum exchange between acoustic and optical waves, i.e., the acoustic wave acts simply as a mirror.

Although there are other SBS-based isolation schemes that deal with higher-order modes (*30*, *31*), they rely on linear mode conversion or intermodal Brillouin scattering and only operate for the fundamental mode. In contrast, isolation of vortex modes is easily realized in chiral PCF just by launching a control wave with the correct topological charge and spin. We note that a nonchiral annual core fiber (*32*) and, most recently, a nonchiral step-index multimode fiber (*33*) can show vortex isolation, although only between modes with different values of |𝓁_A_| since there is no twist-induced birefringence.

Last, we mention that the Brillouin gain would be some 100 times higher if nonsilica glasses such as chalcogenides As_2_Se_3_ or As_2_S_3_ (*34*) were used instead of silica. Although these fibers are challenging to fabricate, they would permit efficient vortex isolation at much shorter (~1-m) fiber lengths. In addition, if implemented using short control pulses, then light-driven vortex isolators could be useful in many different all-optical systems (*10*) and in vortex lasers that are used in optical trapping, communications, and quantum entanglement.

## MATERIALS AND METHODS

### Experimental setup for Stokes wave measurement

The CW light is amplified in an erbium-doped fiber amplifier (EDFA), and the polarization state and spatial phase are controlled using a combination of circular polarizer (linear polarizer and quarter–wave plate) and Q plate. A flip mirror was used to switch the Stokes signal between two paths. In the first path, a circular polarizer was used to measure the Stokes polarization state, and a near-field Brillouin scanning analyzer (NBA) comprising an *xy*-scanning stage, narrow-band notch filter (6 GHz), and power meter was used to precisely monitor the Stokes mode profile and power and to eliminate Fresnel reflections and Rayleigh scattering. In the second path, the Stokes signal was split into two; one half was spatially filtered in a single-mode fiber to produce a divergent near-Gaussian beam, which was then superimposed on the other half, resulting in spiral patterns of fringes related to the topological charge. These patterns were imaged using a charge-coupled device camera after filtering out any stray pump light with a narrow-band filter. This technique was also previously described in (*35*).

### Near-field Brillouin scanning analyzer

This system contains an objective lens, an *xy*-fiber raster scanning stage that is automatically controlled by computer with a close-loop feedback, a narrow-band filter (6 GHz), and signal detection equipment (e.g., power meter, optical spectrum analyzer, and heterodyne system). The Brillouin-shifted signal light, which contains small amounts of pump light (caused by Fresnel reflections or Rayleigh scattering), is collected pixel by pixel by a fiber raster scanning stage. The signal is then filtered to remove the pump light and detected and analyzed by the abovementioned detection equipment. The scanning area is 12 μm × 12 μm and contains 24 × 24 pixels (each pixel area is 0.25 μm^2^). To increase the resolution of the near-field imaging, we used a highly nonlinear fiber with a core diameter of 2.4 μm and numerical aperture of 0.41 (Nufern UHNA7).

### Experimental setup for reconfigurable vortex isolator

The CW laser light at 1550 nm is divided at a fiber coupler. One part is amplified using an EDFA and split into signals in the forward and backward directions using a second fiber coupler. The other part is frequency down- or up-shifted using an electro-optic SSBM and acts as the control wave. The circular polarization states of all the different waves are independently controlled by adjusting the fiber polarization controllers in each optical path, and the optical vortices are optionally generated by Q plates. The setup in the colored frame in Fig. 5A is the key part of nonreciprocal system: The polarization state in each path can be sequentially converted from circular to linear and from linear to circular using two λ/4 plates, and the control signal can be combined with or separated from the forward and backward signal waves using a PBS. After interacting with the control wave, the forward and backward signal waves are extracted using BSs and measured by the NBA systems. The chiral fiber acts as a nonreciprocal isolator when *f*_ctrl_ = *f*_sig_ − *f*_SBS_ and acts as a nonreciprocal amplifier when *f*_ctrl_ = *f*_sig_ − *f*_SBS_.
