# Supplementary material for: Nonreciprocal vortex isolator via topology-selective stimulated Brillouin scattering
Source: Sci Adv. 2022 Oct 19;8(42):eabq6064. doi: 10.1126/sciadv.abq6064 (PMC9581476; doi:10.1126/sciadv.abq6064)
Supplement: Supplementary file 1 — Supplementary Text Figs. S1 to S6 [file sciadv.abq6064_sm.pdf]

Science Advances
AAAS

Supplementary Materials for

Nonreciprocal vortex isolator via topology-selective stimulated Brillouin scattering

Xinglin Zeng *et al.*

Corresponding author: Xinglin Zeng, xinglin.zeng@mpl.mpg.de; Birgit Stiller, birgit.stiller@mpl.mpg.de

Sci. Adv. **8**, eabq6064 (2022)
DOI: 10.1126/sciadv.abq6064

This PDF file includes:

Supplementary Text
Figs. S1 to S6

Supplementary Text

1. Heterodyne setup for Brillouin frequency measurement

A special-designed Brillouin heterodyne setup was used to measure the Brillouin frequency in chiral PCF, as shown in Fig. S1. It is based on heterodyne detection in which the backscattered Stokes light from the PCF is coherently coupled with a local oscillator (LO) to get a beat note in the electrical domain. Both pump and LO are derived from a narrow linewidth (<1 kHz) 1550 nm continuous wave (CW) laser using a fiber coupler. The pump wave is then boosted in an Erbium-doped fiber amplifier (EDFA) and injected into the chiral PCF via an optical circulator. The circular polarization states are adjusted by a fiber polarization controller (FPC) placed before a circulator. The vortex generating module (polarizer, λ/4 plate and Q-plate) is optionally used to generate a circularly-polarized vortex-carrying pump signal. The noise-seeded Stokes signal from the PCF is delivered by the circulator and interferes with the LO using the second 90:10 fiber coupler. Narrow-band (6 GHz) notch filters in the path of Stokes signal are used to filter out Fresnel reflections and Rayleigh scattering. The resulting beat-note is detected in the radio-frequency domain with a fast photodiode (PD) and the averaged Brillouin spectra is recorded with an electrical spectrum analyzer.

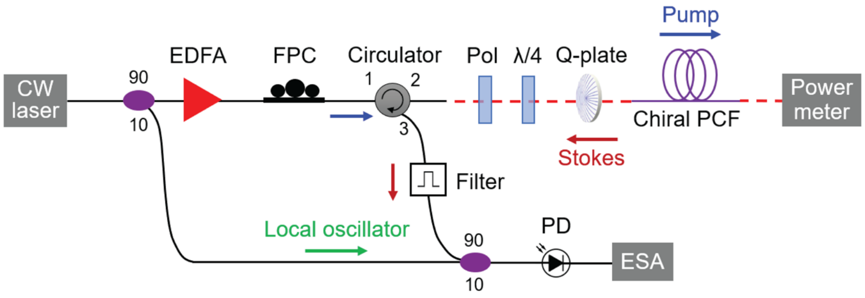

Fig. S1. Experimental setup for measuring spontaneous Brillouin spectra in chiral PCF. A circularly polarized pump signal is generated using a fiber polarization controller (FPC) and a circularly-polarized vortex-carrying pump signal is generated with both an FPC and a vortex generating module. EDFA: Erbium-doped fiber amplifier, Pol: polarizer, λ/4: λ/4 plate, PD: photodetector, ESA: electrical spectrum analyzer.

2. Acoustic modes for Brillouin scattering in chiral PCF

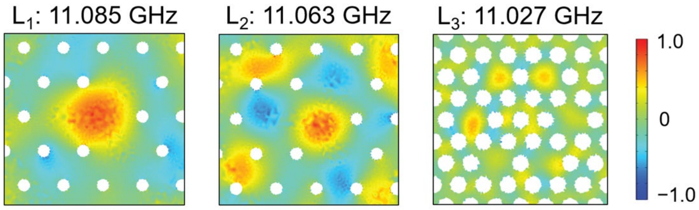

Fig. S2. The numerically calculated axial displacement (normalized to the square-root of the power) of acoustic modes at 11.085 GHz, 11.063 GHz and 11.027 GHz, corresponds to 11.066 GHz, 11.054 GHz and 10.99 GHz Brillouin peak frequencies in Fig. 1d in main manuscript.

3. SBS measurement for [0, ±1] modes in chiral PCF

In this section, we show Stokes power measurements for a circularly polarized LP_{01}-like [0, ±1] pump. Fig. S3a shows the setup. As in all the experiments, the PCF was 200 m long. CW pump light was amplified in an EDFA and its polarization state is controlled using a combination of

polarizing beam splitter (PBS) and λ/4 plate. Back-scattered signals with the same polarization state as the pump are transmitted by the PBS and detected by a power meter placed at port 3 of the circulator, while orthogonally polarized light is reflected and detected by a second power meter. The transmitted pump power is monitored by a third power meter. Narrow-band (6 GHz) notch filters in the path of each Stokes signal are used to filter out Fresnel reflections and Rayleigh scattering. Fig. S2b shows the power dependence of the Stokes and transmitted pump signals for $[0, +1]_P$ (LCP), $[0, -1]_P$ (RCP) and $[0, +1]_P + [0, -1]_P$ (linearly polarized light). Above a threshold of ~1.1 W (independent of the pump polarization state), the orthogonally polarized Stokes signal grows rapidly with a slope efficiency of ~60%, while the transmitted pump power saturates. The co-polarized Stokes signal shows no gain, as angular momentum conservation would otherwise be violated. More interestingly, when a linearly polarized pump is injected into the chiral PCF, the polarization state of the backscattered Stokes wave is also linearly polarized and has same azimuthal angle as that of the pump, as shown in the most right one of Fig. S3b. We attribute this to well-controlled optical activity in the chiral PCF, which causes the linearly polarized pump and backward Stokes modes to be co-polarized at all points along the fiber [29].

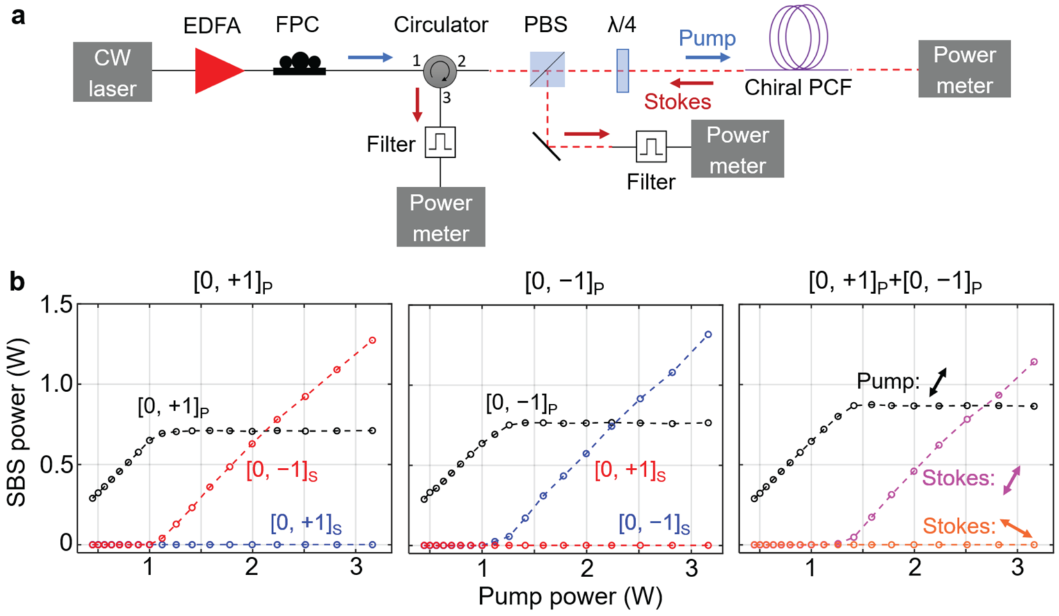

Fig. S3. Stokes measurement in PCF C_3 with [0, ±1] mode pump. a, Experimental setup for measuring SBS power (threshold) for circularly polarized pump in chiral PCF. **b**, Stokes and transmitted pump power in a 200 m length of chiral PCF for $[0, +1]_P$, $[0, -1]_P$ and linearly polarized pump light ($[0, +1]_P+[0, -1]_P$).

4. Stokes parameters $|S_3|$ measurement for Stokes wave in chiral PCF

To assess the robustness of topology- and polarization-selectivity in chiral SBS, the signal powers and Stokes polarization states were measured for $[\ell_T, +1]$ and $[\ell_T, -1]$ pump light. The fiber was spooled with a diameter of 16 cm in all the measurements. In the measurements, the power of the [0, ±1] and [±1, ±1] pump signals entering PCF C_3 was kept at 3.162 W and the power of the [±2, ±1] pump signals entering PCF C_6 was 1.26 W. The modulus of the Stokes parameter S_3 of backscattered Stokes wave, defined as:

$$|S_3| = \left| \frac{P_{RC} - P_{LC}}{P_{RC} + P_{LC}} \right|, \tag{2}$$

ranges in value from 0.96 to 0.99 in the experiments, showing that circular polarization states very well preserved.

5. Measurement of Brillouin gain coefficient

The Brillouin gain coefficient g_B and spectrum were obtained using a typical pump-seed setup with polarization control, as shown in Fig. S4a. Both pump and seed were derived from a narrow linewidth 1550 nm CW laser, the seed light being frequency tuned using a single side-band modulator (SSBM). The pump signal was boosted by an EDFA and the polarization states of both pump and seed were controlled using fiber polarization controllers (FPCs). Vortex generating modules (circular polarizer, Q-plate and λ/2 plate) were optionally used to generate circularly-polarized vortex-carrying pump signals. After propagating backwards through the chiral PCF, the seed signal is reflected by a beam splitter (BS) and filtered by a circular polarizer (polarizer and λ/4 plate). Th flip mirror is used to deliver the Stokes light to either a narrow-band filter and power meter for gain coefficient measurement on [0, ±1] modes or an NBA system (for more details see "Methods" in main manuscript) for gain coefficient measurement on [±1, ±1] modes and [±2, ±1] modes. Fig. S4b shows the measured Brillouin gain spectrum for [0, ±1] modes at a pump power of 0.8 W and a Stokes seed power of 10 mW. The peak gain coefficient is 0.169 $W^{-1}m^{-1}$ for [1, +1] pump and the tiny difference in peak gain in the [0, +1] and [0, −1] pump cases is attributed to slight circular dichroism in propagation loss.

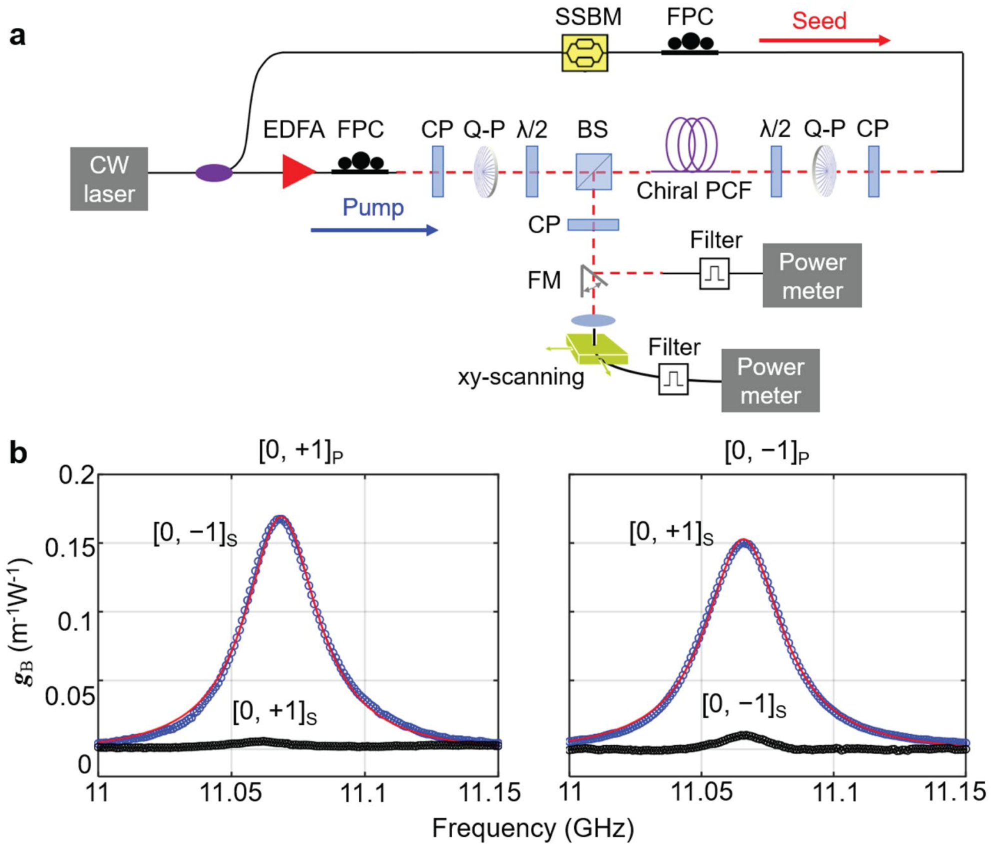

Fig. S4. Brillouin gain spectra measurement in chiral PCF. a, Experimental setup for measuring Brillouin gain spectra of chiral PCF. **b,** The measured Brillouin gain spectra when pumping with $[0, +1]_P$ mode (left) and $[0, -1]_P$ mode (right). The circles are measured data, and the red lines are Lorentzian fittings. FM: flip mirror, CP: circular polarizer, Q-P: Q-plate.

6. Measurement of nonreciprocal isolation and amplification for [0, ±1] modes

Fig. S5a shows the isolation of a circularly polarized LP_{01}-like signal $[0, \pm1]_{sig}$ in the C_3 PCF versus control light power. The isolation is as high as 27.5 dB, for signal power 0.617 W and control power 1.78 W. A nonreciprocal circularly-polarized amplifier can also be implemented by switching the control light frequency from $f_0 - f_{SBS}$ to $f_0 + f_{SBS}$. The measured maximum gain is 33 dB, for signal power 0.65 mW and control power 1.78 W. Higher isolation and amplification factors can be achieved for higher control power or longer fiber length. The isolation remains nearly constant (within ~2dB) over a 35 dB dynamic range of signal power for a control power of 1.78 W, as shown in Fig. S5b.

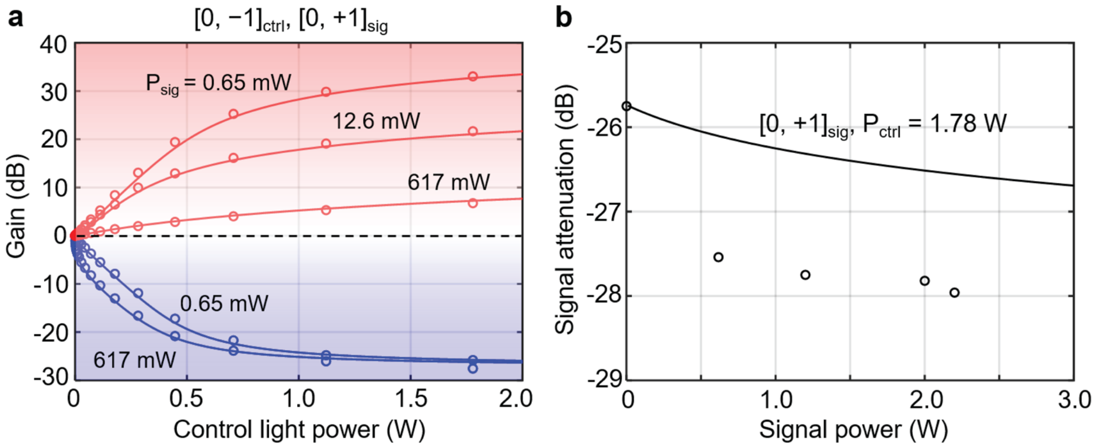

Fig. S5. Nonreciprocal attenuation and amplification for [0, ±1] modes. a, Dependence of attenuation (blue) and amplification (red) of [0, +1] on control light power. **b,** Dependence of signal [0, +1] attenuation on signal power. The circles are experimental datapoints and the curves are theoretical predictions.

7. Experimental setup for the isolation of two vortex modes

The experimental setup is shown in Fig. S6a. CW laser light at 1550 nm is split at a fibre coupler. One part is used as signal and the other as control wave. The first part is delivered either in the forward or the backward direction, and in both cases, the light is split again into two paths to allow generation of different vortex modes. Beam splitters are used to combine and deliver two vortex modes into the isolator. A detailed setup of the signal generator is shown in Fig. S6b. The input CW light is first intensity modulated (IM), then amplified by an EDFA, and finally its circular polarization state and helical phase are set by a polarization controller and a Q-plate. The two signal generators are synchronized.

The second part is frequency down-shifted using an SSBM and amplified in an EDFA. In order to control the signals separately, the control light is also split again into two paths to allow generation of two different vortex modes. The two beams are then combined at a beam splitter.

In this work, the two [−1, +1] and [+1, +1] vortex modes are multiplexed in the forward or backward direction and two CW control waves are multiplexed in the backward direction.

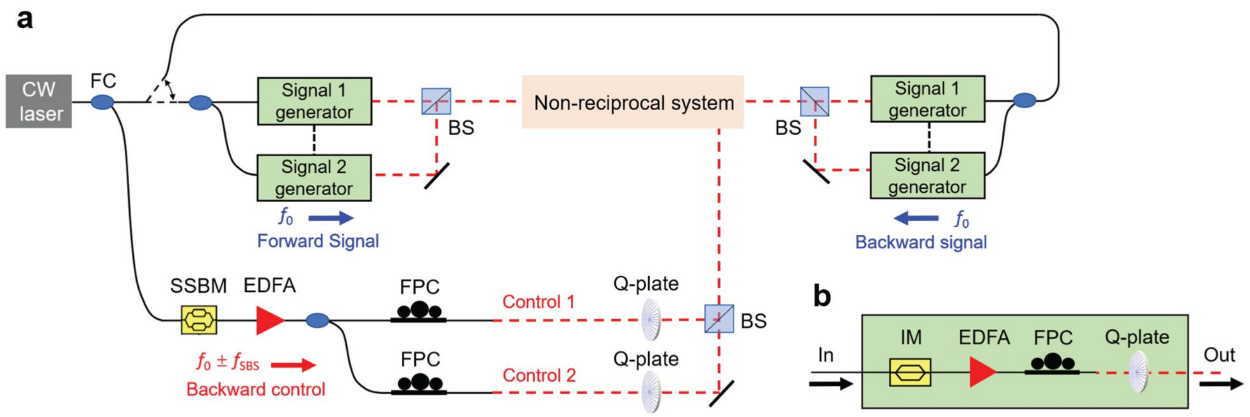

Fig. S6. Experimental setup for two vortex modes isolation. a, Experimental setup. **b,** detailed experimental setup of "signal generator".
